# The Role of the Octarepeat Region in Neuroprotective Function of the Cellular Prion Protein

**DOI:** 10.1111/j.1750-3639.2007.00061.x

**Published:** 2007-04-01

**Authors:** Gerda Mitteregger, Milan Vosko, Bjarne Krebs, Wei Xiang, Veronika Kohlmannsperger, Svenja Nölting, Gerhard F Hamann, Hans A Kretzschmar

**Affiliations:** 1 Center for Neuropathology and Prion Research Munich, Germany; 2 Department of Neurology, Ludwig-Maximilians University Munich, Germany

## Abstract

Structural alterations of the cellular prion protein (PrP^C^) seem to be the core of the pathogenesis of prion diseases. However, the physiological function of PrP^C^ remains an enigma. Cell culture experiments have indicated that PrP^C^ and in particular its N-terminal octarepeat region together with the phosphatidylinositol 3-kinase (PI3K)/Akt signaling pathways have a fundamental involvement in neuroprotection and oxidative stress reactions. We used wild-type mice, PrP knockout (*Prnp*^−/−^) animals and transgenic mice that lack the octarepeat region (C4/−) and subjected them to controlled ischemia. We identified an increased cleavage and synthesis of PrP^C^ in ischemic brain areas of wild-type mice compared with sham controls. The infarct size in *Prnp*^−/−^ animals was increased threefold when compared with wild-type mice. The infarct size in C4/− animals was identical to *Prnp*^−/−^ mice, that is, around three times larger than in wild-type mice. We showed that the PrP in C4/− mice does not functionally rescue the *Prnp*^−/−^ phenotype; furthermore it is unable to undergo β cleavage, although an increased amount of C1 fragments was found in ischemic brain areas compared with sham controls. We demonstrated that the N-terminal octarepeat region has a lead function in PrP^C^ physiology and neuroprotection against oxidative stress *in vivo*.

## INTRODUCTION

The cellular prion protein (PrP^C^) is a copper-binding protein (3, 17) located at the synapse (13) and has a primary role in the pathogenesis of prion diseases. A templated conformational transition of PrP^C^ seems to be the cause of transmissibility and pathogenesis (25). PrP^C^ has been shown to play a role in cellular antioxidative defense mechanisms (15, 27) and to protect human neurons in primary culture against Bax-mediated cell death (2). Experiments in cerebellar granule cells (5) have shown a loss of neuroprotective activity of PrP^C^ by a deletion of all five N-terminal octarepeats (PrPΔ51–90). We have recently identified a functional link between PrP^C^ expression and phosphatidylinositol 3-kinase (PI3K) activation, a regulator of Akt phosphorylation and a protein kinase that plays a pivotal role in cell survival (33). Our findings are in agreement with published results that showed reduced Phospho-Akt expression levels in *Prnp*^−/−^ mouse brains following ischemia (38). We could also demonstrate that hippocampal cells that lack the octarepeat region showed reduced PI3K levels and decreased survival under stress conditions similar to PrP^C^ knockout cells.

In neuroblastoma cells it could be demonstrated that the octapeptide repeats are required for the β cleavage and their absence (PrPΔoct) correlates with increased sensitivity of cells to oxidative stress (36). The cleavage of the molecule seems to play a major role in the biological properties of PrP^C^ but succession of these proteolysis procedures as well as relations between the two cleavages is controversial and still remains to be elucidated and in particular, the relevance of the octapeptide region in this functional process has not been established *in vivo*.

Recently, brain injury models in mice have been used to investigate a potential neuroprotective role of PrP^C^ *in vivo* (14, 21, 31, 37). There were significant differences between the volume of the lesion in PrP knockout (*Prnp*^−/−^) mice when compared with the wild-type counterpart with larger area of injury in the former. Moreover, using *in situ* hybridization an increase of PrP mRNA levels and also PrP immunoreactivity were detected in penumbral neurons of the ischemic lesion (21). These results further support the hypothesis of the antioxidant function of PrP^C^ *in vivo*.

With this work we specify the neuroprotective property of PrP^C^ and the role of the octarepeat region in this function and contribute to understanding the succession and significance of cleavage processes in an *in vivo* ischemic model. We used transgenic C4/− mice that lack the octarepeat region of PrP^C^ (6). We challenged C4/−, *Prnp*^−/−^ and *Prnp*^+/+^ mice in a focal cerebral ischemia model. *Prnp*^−/−^ and C4/− mice showed largely increased infarct volumes when compared with wild-type littermates. The C4 protein (PrPΔ32–93) did not functionally rescue the *Prnp*^−/−^ phenotype, the octapeptide region of PrP^C^ therefore seems to play a key role in neuroprotection.

## MATERIALS AND METHODS

### Transgenic mice

We used homologous *Prnp*^+/−^ recombination to produce *Prnp*^+/+^ and *Prnp*^−/−^ littermates with a 129/Sv-C57/Bl6 background. The role of the octarepeat region was investigated in C4/− mice lacking amino acids 32 to 93 of PrP^C^ (30). In consideration of the *Prnp*^−/−^ background of C4/− mice and the PrP^C^ expression, we crossed hemizygous C4/− and *Prnp*^−/−^ mice to obtain male (21–28 g) hemizygous C4/− and *Prnp*^−/−^ littermates. Published polymerase chain reaction (PCR) protocols to identify the transgenes on the *Prnp*^−/−^ background or wild-type and *Prnp*^−/−^ mice were used (30). In agreement with previous findings published by Shmerling and coworkers (30), we detected higher PrP expression in C4/− animals in comparison with wild-type control mice, that is, approximately three times higher in homozygous and 1.5 times higher in hemizygous C4/− mice (data not shown). All C4 mice used in this study were hemizygous C4/−. The mice were assigned to four groups: G1 (*Prnp*^−/−^; n = 9), G2 (*Prnp*^+/+^; n = 7), G3 (C4/−; n = 8), G4 (*Prnp*^−/−^; n = 8). They were kept under standard diurnal conditions and allowed access to food and water *ad libitum*. The animal experiments were in accordance with animal protection standards and were approved by the government of Upper Bavaria (protocol number 209.1-2531-81/01). Every effort was made to reduce the number of animals used and to ensure they were free of pain and discomfort.

### Surgical procedure

The intraluminal filament model was used for occlusion of the middle cerebral artery (MCAO) (35). After 1 h of ischemia reperfusion was induced by withdrawing the thread, the animals were then sacrificed after 24 h. Briefly, mice were anesthetized with 3% isoflurane (Forene, Abbott, Germany) for induction and maintained on 1.5% in a 70%/30% mixture of N_2_O/O_2_ administered via a face mask. The rectal temperature was maintained at 37 ± 0.5°C with a homeothermic blanket system. The regional cerebral blood flow (rCBF) was recorded in all animals using laser Doppler flowmetry (Perimed, Järfäla, Sweden) in which a fiberoptic probe placed over the MCA territory 2 mm posterior and 6 mm lateral of the bregma (12). After ventral midline neck incision, the left common and external carotid arteries were ligated. The internal carotid artery (ICA) was temporarily clipped with a microvascular clip (Aesculap, Tuttlingen, Germany), and a silicon-coated 8-0 nylon monofilament (Ethicon, Johnson&Johnson, Norderstedt, Belgium) was gently advanced into the ICA. The baseline rCBF values were reduced by >70%. The tip diameter of the thread was selected to match the body weight of the animals. After surgery, the laser Doppler probes were removed, and mice were allowed to wake up and return to their cages. Reperfusion was initiated by withdrawing the thread under short inhalation anesthesia. At the end of the reperfusion period, the mice were sacrificed in deep anesthesia by transcardial perfusion with cold isotonic saline solution containing bovine serum albumin (5 g/L), heparin (10 IU/L) and 2 mL nitroprusside sodium (100 µL, 18 mg in 10 mL saline) solution. Brains were removed, and the skull base was inspected for hemorrhage. In addition, four sham-operated mice underwent the same surgical procedures, but in these mice the filament was only introduced into the common carotid artery; this does not lead to brain infarction. The neurological outcome was defined in all animals according to Garcia et al (7). It is defined by a sequence of six physiological tasks: (i) spontaneous activity, (ii) symmetry in the movement, (iii) forepaw outstretching, (iv) climbing, (v) body proprioception, and (vi) response to vibrissae touch. The overall score is 18 points. In all surgical procedures and following experiments the investigators were blinded as to the experimental groups individual mice belonged to.

### Preparation of frozen sections

**Figure 1:**
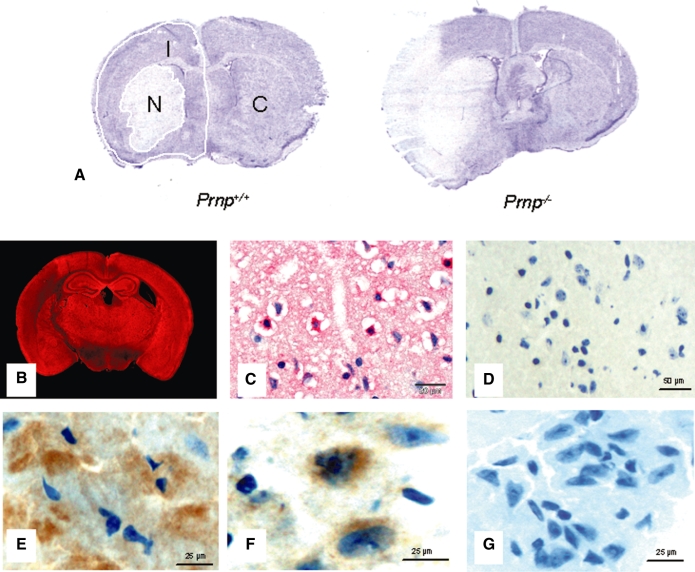
Histology after middle cerebral artery occlusion for 1-h ischemia and 24-h reperfusion time **A.** Nissl-stained sections at the level of the bregma, the maximal extent of the infarct in *Prnp*^−/−^ and *Prnp*^+/+^ mice. Necrotic areas show a very faint staining. Frozen coronal sections were divided into three regions, that is, necrotic infarct area (N), ipsilateral non-necrotic hemisphere (I) and contralateral hemisphere (C). **B.** Immunofluorescence for cellular prion protein (PrP^C^) in a *Prnp*^+/+^ mouse after cerebral ischemia using polyclonal antibody CDC1 and Fast Red as chromogen. There is strong staining for PrP^C^ in all gray matter areas except the infarct zone on the left. **C.** Immunohistochemistry with polyclonal antibody CDC1. This formalin-fixed and paraffin-embedded section shows a number of strongly stained neurons in the area adjacent to the infarct, while other neurons and glial cells in this section appear negative. **D.** No reaction with the antibody directed against PrP was detected in frozen sections of the *Prnp*^−/−^ littermates. **E.** Immunofluorescence for PrP^C^ in *Prnp*^+/+^ mouse brains after cerebral ischemia using polyclonal antibody CDC1 and DAB as the chromogen. This frozen section shows again a number of strongly stained neurons in the area adjacent to the infarct. **F.** Immunofluorescence for PrP^C^ in C4/− mouse brains after cerebral ischemia using polyclonal antibody 1A8 and DAB as the chromogen. Punctate PrP staining of intensely immunoreactive neuronal cells is seen especially in the ipsilateral area surrounding the infarct. **G.** No reaction with the antibody 1A8 directed against PrP was detected in the frozen sections of the *Prnp*^−/−^ littermates.

Frozen sections of 10-µm thickness were collected on pre-labeled glass slides at −20°C. Every 50th section was used for volumetric analysis; the remaining sections were stored at −80°C. For Western blotting and reverse transcriptase polymerase chain reaction (RT-PCR), frozen coronal sections were prepared, Nissl-stained and divided using a scalpel and a dissecting microscope into three regions, that is, necrotic infarct area (N), ipsilateral non-necrotic hemisphere (I), and contralateral hemisphere (C) to detect different progressions of the damage in dependence on the injury distance (Figure 1A).

### Infarct definition and volumetry

Nissl staining was used to delineate infarct lesions. To calculate the infarct and hemispheric volumes, the method described by Kloss et al (16) was used. Briefly, Nissl-stained consecutive sections with 500-µm clearance were digitized using a flatbed scanner (Epson, Meerbusch, Germany). The files were imported into Optimas imaging software, the lesions and hemispheres delineated, and the size converted into the metric system. Next, partial volumes between two adjacent sections were computed using the formula for a conic section: 
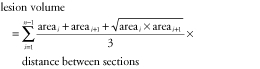
 with area _1,2,3, … n−1_ being the partial area of the lesion of the serial sections. The total hemisphere volumes were calculated equally and finally the ratio of infarct volume vs. the total hemispheric volume was defined.

### Quantitative RT-PCR (QRT-PCR)

Immediately prior to RNA isolation, slides containing frozen sections were fixed in 75% ethanol for 45 s and Nissl-stained. Subsequently, the slides were dehydrated in graded ethanol solutions for 5 s each and air-dried. RNA isolation from Nissl-stained sections was performed by using RNeasy Micro Kit (Qiagen, Hilden, Germany). Briefly, materials in particular brain regions (N, I and C) as described above were scraped and lysed with buffer RLT. Materials in a particular region from eight sections each animal were pooled and RNAs isolated according to the protocol recommended by Qiagen. The quantity of RNA was assessed using the NanoDrop® ND-1000 Spectrophotometer (NanoDrop, Wilmington, DE, USA).

Four hundred nanograms of RNA was used to make single-stranded cDNAs (Superscript II; Invitrogen, Karlsruhe, Germany) according to the manufacturer’s instructions. Using 2 µL diluted cDNA (1:10), real-time quantitative PCR was carried out with Faststart Plus DNA Master SYBR Green I on LightCycler (Roche, Mannheim, Germany) under the following conditions: 95°C for 10 minutes for a hot start, followed by denaturing at 95°C for 10 s, annealing at 57°C for 5 s, and extension at 72°C for 10 s for 45 cycles. Primer sets used for PCRs were as follows: for β-actin (9) (with modification), 5′ AAC CCT AAG GCC AAC CGT GAA AAG 3′ and 5′ CTA GGA GCC AGA GCA GTA ATC T 3′; for prion protein gene (*Prnp*), 5′ CCA ATT TAG GAG AGC C 3′ and 5′ GCT TCC CTG CCC GGG ATA CC 3′. Calibrator-normalized relative quantification was performed by LightCycler Software version 4.0 (Roche).

### Immunohistochemical staining procedures

Coronal frozen sections were pretreated including dehydration, short fixation in 0.25% glutaraldehyde and heat-reliant epitope retrieval. Afterwards, nonspecific protein binding sites were blocked with 10% normal goat serum followed by incubation of the respective primary antibody. For PrP staining in *Prnp*^+/+^ mice, an antiserum purified from a rabbit immunized against recombinant full-length mouse PrP was used (CDC1), whereas 1A8, an antiserum purified from a rabbit immunized against the C-terminal domain (amino acids 89–234) of mouse PrP was used in C4/− mice. The polyclonal antibody Actin I-19 against β-actin was obtained from Santa Cruz (Biotechnology, Heidelberg, Germany). The primary antibody was detected by a biotinylated secondary antibody and enhanced by streptavidin alkaline phosphatase complexes. Visualization of immunoreactivity followed by using Fast Red or DAB as the chromogen. Counterstaining followed with hemalaun. For immunofluorescence staining, a streptavidin CY3 conjugate was used instead of streptavidin alkaline phosphatase complexes. The nuclei were counterstained with 4′,6-diamidino-2-phenylindole dihydrochloride and slides were covered in aqueous solution. Staining of paraffin sections was performed after deparaffinizing the sections in xylene.

### Immunoblotting

The process was performed as described in our previous data (18). According to the size, different regions (N, I and C) on the Nissl-stained coronal frozen sections were solubilized by adding 5 µL of lysis buffer (with 2% SDS and 0.5 M DTT) on N, 10 µL on I and 15 µL on C. Identical regions were solubilized in the same volumes of buffer in sham-operated animals. In five animals without surgery the brains were perfused to obtain calibration measurements. The solubilized tissue was transferred to reaction tubes and digested with PNGase according to the standard protocol of the manufacturer (Roche Diagnostics, Penzberg, Germany). Digestion was stopped by the addition of modified Laemmli sample buffer and boiling for 10 minutes. Five microliters of each sample was subjected to Western blotting. Proteins were separated on 12% SDS-PAGE (NuPAGE, Invitrogen, Karlsruhe, Germany) and transferred to PVDF membranes (Millipore, Eschborn, Germany). After short blocking, the membranes were incubated with the primary antibodies, 6H4 (Prionics, Schlieren, Switzerland) and Actin I-19 (Biotechnology, Heidelberg, Germany), separately, over night. After incubation with primary antibody, the membranes were incubated with secondary antibodies, coupled to alkaline phosphatase (DakoCytomation, Hamburg, Germany).

The complete process was performed with exact timing of the individual steps and at a constant temperature to reach maximum accuracy. For quantification of relative signal intensities, each Western blot contained a dilution series of a standard protein with known concentration. Intensity measurements were done with Total Lab V 2.01 software (Nonlinear Dynamics, Newcastle-upon-Tyne, UK). The Western blots of actin were used as a reference.

### Statistical analysis

Statistical analysis was performed using the SPSS software system (SPSS for Windows, Version 9.0, SPSS, Inc., Chicago, IL, USA). The parametric T-test for unpaired samples was used to determine significant differences between two groups. All *P*-values were two sided and *P* < 0.05 was considered significant.

## RESULTS

### Characteristics of the ischemic brain injury in *Prnp^+/+^* and *Prnp^−/−^* mice

Nissl-stained sections from the brains subjected to 1-h MCAO and 24-h reperfusion were examined. Infarcts were clearly demarcated in all experimental groups. The damaged areas appeared as pale areas on gross inspection (Figure 1A). Severe swelling of the affected hemisphere with a corresponding midline shift was noticed. Clearly delineated lesions in the ischemic hemisphere were found in all animals after 24 h of reperfusion. Ischemic lesions extended from the forebrain and few millimeters past the bregma with the maximum extent of the lesion at the level of the bregma.

**Figure 2:**
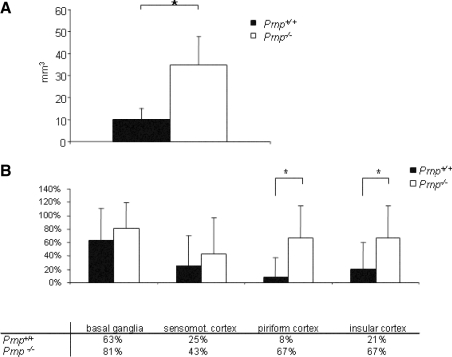
**A.** Lesion volumes of *Prnp*^+/+^ and *Prnp*^−/−^ mice after 1 h of ischemia and 24-h reperfusion. *Prnp*^−/−^ mice (n = 9) show a more than three times larger lesion volume than *Prnp*^+/+^ mice (n = 7). Data are means ± standard deviation. *P < 0.05 (Student’s *t*-test). **B.** Neuroanatomical pattern of necrosis in *Prnp*^+/+^ and *Prnp*^−/−^ mice. The graph demonstrates the extent of the infarct over various anatomical regions in the ischemic hemisphere at the level of the bregma. The affected areas in the piriform and insular cortex were significantly larger in *Prnp*^−/−^ than in *Prnp*^+/+^ mice (*P < 0.05). Error bars indicate standard errors of the mean; mean values are given in the table below.

Lesion volumes, measured by summarizing delineated infarct areas in 500 µm steps, were 10.06 mm^3^ ± 4.8 mm^3^ in *Prnp*^+/+^ mice and 34.78 mm^3^ ± 12.99 mm^3^ in *Prnp*^−/−^ mice (Figure 2A). The partial volumes of the hemispheres were measured from −3.0 mm to +7.0 mm behind the bregma, and were 50.19 mm^3^ ± 19.5 mm^3^ in the ipsilateral hemisphere and 50.89 mm^3^ ± 19.88 mm^3^ in the contralateral hemisphere in *Prnp*^+/+^ mice. In *Prnp*^−/−^ mice they were 86.45 mm^3^ ± 18.4 mm^3^ in the ipsilateral and 79.20 mm^3^ ± 15.03 mm^3^ in the contralateral hemisphere. Thus there was a much more pronounced increase in volume in both hemispheres in *Prnp*^−/−^ than in *Prnp*^+/+^ mice. The ratio of the infarct volume to the ipsilateral hemisphere volume showed an infarction of 39% ± 10% in *Prnp*^−/−^ mice and 20% ± 3% in *Prnp*^+/+^ mice. These differences were significant between *Prnp*^+/+^ and *Prnp*^−/−^ mice (*P* < 0.05).

### Anatomical distribution, appearance, size and pattern of the infarct

During evaluation of the infarct size we noticed that various anatomical regions were affected in different degrees in *Prnp*^+/+^ and *Prnp*^−/−^ mice. A more detailed comparison of sections at the level of the bregma (Figure 2B) showed that the basal ganglia were most consistently and the most severely affected; the area affected was 63% in *Prnp*^+/+^ mice while it was 81% in *Prnp*^−/−^ mice. Twenty-five percent of the motor cortex area was affected in *Prnp*^+/+^ and 43% in *Prnp*^−/−^ mice. The highest diversity was found in the piriform cortex with 8.3% in *Prnp*^+/+^ and 67% in *Prnp*^−/−^ mice followed by the insular cortex with 21% in *Prnp*^+/+^ and 67% in *Prnp*^−/−^ mice. The differences in the piriform cortex and the insular cortex of *Prnp*^+/+^ and *Prnp*^−/−^ mice were statistically significant (*P* < 0.05). Only 7.1% of the motor cortex was affected in *Prnp*^−/−^ mice, whereas the cingulate cortex was unaffected in *Prnp*^+/+^ as well as in *Prnp*^−/−^ mice. There were no pathologic findings in paraventricular nuclei. In conclusion, the greatest variability was seen in the piriform and the insular cortex.

### Neurological evaluation

**Figure 3:**
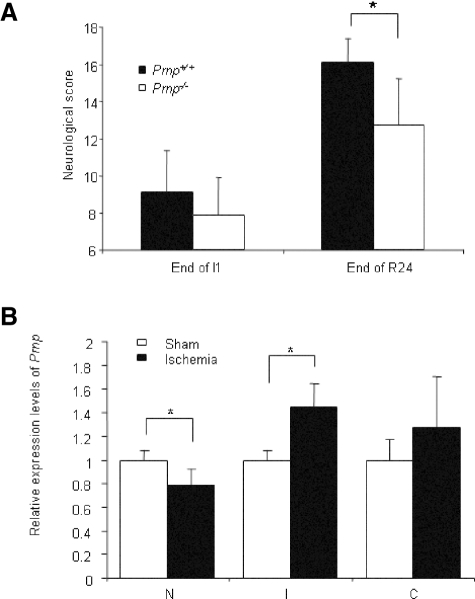
**A.** Neurological scores (±standard deviation) in *Prnp*^+/+^ and *Prnp*^−/−^ mice subjected to transient middle cerebral artery occlusion after 1 h of ischemia (End of I1) and at the end of the 24-h lasting reperfusion period (End of R24). No significant differences were seen after 1 h of ischemia, although there was a tendency for *Prnp*^−/−^ mice to perform less well. After 24-h reperfusion the scores of *Prnp*^−/−^ mice were significantly lower than in *Prnp*^+/+^ mice. The asterisk indicates significant difference (*P* < 0.002) as determined by *t*-test. **B.** Quantitative reverse transcriptase polymerase chain reaction analysis of *Prnp* gene transcription after 1 h of transient focal cerebral ischemia and 24-h reperfusion. The relative transcription levels of *Prnp* and β-actin genes in the regions containing the necrotic infarct area (N) and the region free of infarct of the ipsilateral hemispheres (I) as well as the expression levels in the contralateral hemispheres (C) in ischemic animals are indicated by fold-changes as compared with the corresponding regions of sham-operated animals (n = 4 for all groups). The asterisk indicates a significant increase in *Prnp* transcription in the ipsilateral hemisphere I and a significant decrease in the necrotic area N compared with sham-operated animals as determined by Student’s *t*-test (*P* < 0.05). There is a tendency for increased *Prnp* transcription in the contralateral hemisphere C, which is statistically not significant.

The neurological examination was carried at two different times, after 1 h of ischemia and at the end of the 24-h reperfusion period. The correlation between the mean neurological score and mean number of necrotic neurons after focal cerebral ischemia has been described as highly significant (7); a decrease of the score stands for an increase of clinical symptoms. A mean neurological score of 18 points represents a healthy mouse without clinical symptoms. The mean neurological scores after 1 h of ischemia were significantly lower than those after 24 h of reperfusion in *Prnp*^+/+^ and *Prnp*^−/−^ mice (Figure 3A). While after 1 h of ischemia no differences were noted between *Prnp*^−/−^ and *Prnp*^+/+^ mice, after 24-h reperfusion the scores of *Prnp*^−/−^ mice were significantly lower compared with *Prnp*^+/+^ mice (*P* < 0.002).

### RT-PCR analysis of *Prnp* transcription after transient focal cerebral ischemia

Following 1-h transient focal cerebral ischemia and 24-h reperfusion in *Prnp*^+/+^ animals three distinct areas were subjected to investigation: (N) the region containing the necrotic infarct area, (I) the region free of infarct in the ipsilateral hemisphere and (C) the region free of infarct in the contralateral hemisphere. Transcription levels of *Prnp* from these areas were analyzed by QRT-PCR and were compared with the corresponding ipsilateral (I) and the contralateral hemispheres (C) of sham-operated animals (Figure 3B). RT-PCR analysis revealed a significantly increased expression of *Prnp* in the infarct-free region of the ipsilateral hemisphere after ischemia (*P* < 0.05). *Prnp* expression showed a slight but nonsignificant increase in the contralateral hemisphere. In contrast, PrP^C^ mRNA levels were significantly decreased in the necrotic area (N) (*P* < 0.05), which may be associated with the massive cell death in this area.

### Histology and immunohistochemistry

In the Nissl-stained sections the nerve cells in the infarct region showed pale staining (Figure 1A). They appeared shrunken with small pyknotic nuclei. Vacuolar changes were also frequently observed. The polyclonal rabbit antibody directed against recombinant full-length mouse PrP (CDC1) reacted strongly in neurons and sometimes glial cells in both gray and white matter (Figure 1B). In gray matter areas single strongly immunoreactive neurons were observed predominantly in the hippocampus and neocortex in MCAO and control animals. In the necrotic infarct area there was no PrP^C^ immunostaining. In contrast, numerous neuronal cells in the area surrounding the infarct region in the ipsilateral hemisphere (I) showed a strong PrP^C^ immunoreactivity, while other neurons in the immediate vicinity did not react (Figure 1C). Sections of *Prnp*^−/−^ mice showed no reaction with the antibody directed against PrP^C^ (Figure 1D).

### Western blotting

Western blots were performed with tissue homogenates from brain sections at the level of the bregma. By using the primary antibody 6H4, which is directed against an epitope within the first alpha helix, full-length unglycosylated PrP^C^ (PrP^C^ FL) was detected at an apparent molecular weight of 28.5 kDa. Two additional fragments were observed at about 21.1 and 16.4 kDa corresponding to the C2 and C1 fragments described by Mangé (20). Reprobing of Western blots with a primary antibody against β-actin showed a band at 42.8 kDa.

**Figure 4:**
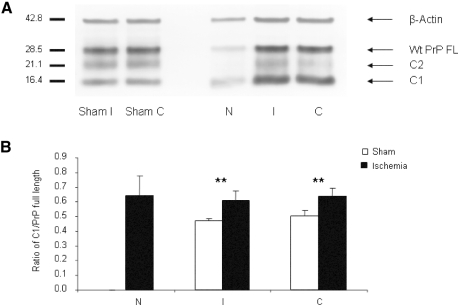
Expression of cellular prion protein *(*PrP^C^) and generation of cleavage products in *Prnp^+/+^*mice **A.** Western blots were performed with PNGase-digested tissue homogenates from the necrotic infarct area (N), infarct-free brain tissue of the ipsilateral hemisphere (I) and contralateral hemisphere (C). A strong band representing full-length PrP^C^ (PrP^C^ FL) was detected at about 28.5 kDa. Additional bands of lower molecular weight can be observed at about 21.1 kDa, corresponding to the C2 fragment, and at 16.4 kDa, corresponding to the C1 fragment of PrP^C^. As a reference the blots were reprobed with an antibody directed against β-actin. PrP^C^ was found decreased in necrotic areas of ischemic mouse brains, it was increased in non-necrotic areas of the ipsilateral and contralateral hemispheres. **B.** Densitometric analysis of the ratio of C1/PrP^C^ full length reveals a significant relative increase of C1 in both the necrotic infarct area (N) and the infarct-free area of the ipsilateral hemisphere (I), and of the contralateral hemisphere (C) in ischemic mouse brains (n = 7) in comparison with areas of sham-operated controls (n = 4). Error bars indicate standard deviations. Significant differences between ischemic and sham controls are marked by an asterisk (**P < 0.01) as determined by Student’s *t*-test.

In the samples extracted from the right and left hemispheres of sham-operated mice (Figure 4A; lanes Sham C and Sham I) PrP^C^ FL represented the strongest signal, whereas bands corresponding to the C1 fragment showed weaker staining. The C2 fragments were weaker but clearly discernible. The expression level of β-actin was similar in both hemispheres. In contrast to this pattern, the necrotic infarct area (N) of an ischemic mouse brain showed weak staining for β-actin. PrP^C^ FL, C1 and the C2 fragments were barely visible. In comparison with PrP^C^ FL the C1 appeared slightly stronger. Strong C1 bands were also seen in the ipsilateral hemisphere not affected by the infarct (I) and the contralateral hemisphere (C) of ischemic mice. These bands were not as intense as the bands corresponding to PrP^C^ FL, but remarkably stronger than those representing the C1 fragment in sham-operated mice.

The detected proteins were quantified via comparison with a dilution series (not shown). Figure 4B demonstrates the analysis of the ratio of C1 to PrP^C^ FL in sham-operated (white columns) and ischemic mice (dark columns). The ratio of the C1 fragment to PrP^C^ FL was significantly increased in ischemic mice compared with sham-operated animals in the ipsilateral hemisphere I and contralateral hemisphere C (*P* < 0.01).

### C4/− (PrPΔ32–93) compared with *Prnp^−/−^* mice

#### Infarct volume

**Figure 5:**
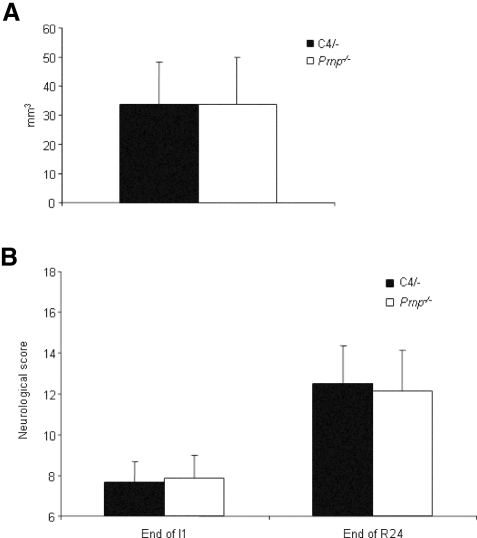
**A.** Lesion volumes of C4/− and *Prnp*^−/−^ mice after 1 h of ischemia and 24-h reperfusion. There are no significant differences between C4/− and *Prnp*^−/−^ mice. Data are means ± standard deviation; n = 8 animals/group. **B.** Neurological scores (±standard deviation) in C4/− and *Prnp*^−/−^ mice subjected to transient middle cerebral artery occlusion after 1 h of ischemia (End of I1) and at the end of the 24-h lasting reperfusion period (End of R24). No significant differences in the neurological score were found at I1 and R24.

Lesion volumes measured by summarizing delineated infarct areas in 500 µm steps were 33.54 mm^3^ ± 14.93 mm^3^ in C4/− mice and 33.85 mm^3^ ± 16.13 mm^3^ in *Prnp^−/−^* mice after 24 h of reperfusion (Figure 5A). The volume of the hemispheres were measured from −3.0 mm to +7.0 mm behind the bregma, and reached 86.44 mm^3^ ± 29.28 mm^3^ ipsilaterally and 76.12 mm^3^ ± 24.66 mm^3^ contralaterally in C4/− mice. The brain sections of *Prnp*^−/−^ mice showed a hemispheric volume of 82.31 mm^3^ ± 26.26 mm^3^ in the ipsilateral hemisphere and 71.44 mm^3^ ± 19.14 mm^3^ in the contralateral hemisphere. The ratio of ipsilateral to contralateral hemisphere was 113% ± 6% in C4/− mice and 114% ± 10% in *Prnp*^−/−^ mice. These results signify a shift to the contralateral hemisphere in C4/− and *Prnp*^−/−^ mice and stand for serious implications of the damage caused by transient MCAO in both strains in a similar manner. The ratio of the infarct area to the ipsilateral hemispheric volume showed infarction with 37% ± 6% in C4/− mice and 39% ± 10% in *Prnp*^−/−^ mice. In conclusion, there were no differences in infarct volume and ratio of infarct volume to the ipsilateral hemisphere between C4/− and *Prnp*^−/−^ mice.

#### Neurological evaluation

The neurological examination of C4/− vs. *Prnp*^−/−^ littermates was carried at two different time points, after 1 h of ischemia and at the end of 24-h reperfusion. The mean neurological scores of C4/− and *Prnp*^−/−^ mice after 1 h of ischemia were significantly lower than those after 24-h reperfusion, no significant difference was seen between the C4/− mice and the *Prnp*^−/−^ mice, neither after 1 h of ischemia nor at the end of 24-h reperfusion (Figure 5B).

#### Histology and immunohistochemistry

In both C4/− and *Prnp*^−/−^ mice, a remarkable swelling of the ischemic hemisphere with midline shift to the contralateral hemisphere was observed in a similar manner. After the 24-h reperfusion period, clearly delineated lesions in the ischemic hemisphere were found in both groups. In C4/− and *Prnp*^−/−^ mice the ischemic lesions began in the forebrain and ended a few millimetres behind the bregma. Furthermore, C4/− and *Prnp*^−/−^ mice showed the maximal extent of the lesion at the level of the bregma. After focal cerebral ischemia the necrotic lesion area in the ipsilateral hemisphere of the C4/− mice showed a remarkably reduced immunoreactivity for PrP and β-actin. The ipsilateral area surrounding the infarct presented a punctate PrP staining of intensely immunoreactive neuronal cells in a pale environment as seen in *Prnp*^+/+^ mice (Figure 1F). Similar to sections of *Prnp*^+/+^ mice, C4/− mice had indicated strongly PrP immunoreactive neurons predominantly in the hippocampal formation and cerebral cortex. An overall weak staining in the corpus callosum and basal ganglia was observed, while most immunoreactivity was found again in the hippocampus and piriform gyrus. In comparison, no reaction with the antibody directed against PrP was detected in the frozen sections of the *Prnp*^−/−^ littermates (Figure 1G). β-Actin immunoreactivity was observed in C4/− and *Prnp*^−/−^ mice overall in an analogous manner. Both strains showed strong staining in the basal ganglia and outer layers of the cortex and weaker staining in the corpus callosum.

#### Western blotting analysis of PrP^C^ after MCAO

**Figure 6:**
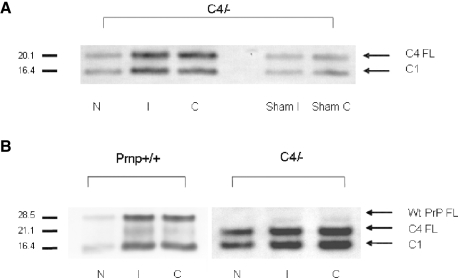
Expression of cellular prion protein *(*PrP^C^) and generation of cleavage products in C4/− and *Prnp^+/+^* mice The Western blots were performed with PNGase-digested homogenates of sham-operated C4/− mice and the three different brain regions of C4/− and *Prnp*^+/+^ mice after ischemic injury (N, I and C). **A.** Dominant bands of C4 full-length (C4 FL) are detected at about 20.1 kDa in C4/− sham control mice. At about 16.4 kDa there are weaker bands that correspond to the C1 fragments in C4/− mouse brain homogenates. After middle cerebral artery occlusion (MCAO) damage in C4/− mice there is an increase in α cleavage in the three regions investigated (N, I, C). Two bands at about 20.1 and 16.4 kDa are seen corresponding to full-length C4 protein and the C1 fragment. The signal intensities of C1 fragments in ischemic C4/− mice are remarkably stronger than those representing the C1 fragment in sham-operated control mice. A C2 fragment is not discernible. **B.** A clear band of full-length PrP^C^ (PrP^C^ FL) is detected at about 28.5 kDa in *Prnp*^+/+^ mice and about 20.1 kDa in C4/− mice. An additional band is seen at 21.1 kDa in the *Prnp*^+/+^ lanes corresponding to the C2 fragment. The C2 fragment of PrP^C^ migrates approximately at the same molecular weight as the full-length C4 protein (PrPΔ32–93). A C2 fragment is not discernible in C4/− mice. At about 16.4 kDa there are dominant bands that correspond to the C1 fragments in *Prnp*^+/+^ and C4/− mouse brain homogenates after MCAO damage.

Figure 6A shows Western blots of PNGase-digested identical brain samples of ischemic C4/− mice (N, I and C) and corresponding areas in C4/− sham control animals with homogenates from brain sections at the level of the bregma. Antibody 6H4 was used as primary antibody, which is directed against an epitope within the first alpha helix of PrP^C^. Only two bands at about 20.1 and 16.4 kDa are seen corresponding to the full-length C4 protein and the C1 fragment, whereas no isolated C2 fragment is discernible. In comparison with C4 FL the C1 fragments appeared slightly stronger in necrotic and in the ipsilateral hemisphere not affected by the infarct (I) after MCAO damage in C4/− mice but a C2 fragment is not detectable in C4/− mice. In the samples extracted from the ipsi- and the contralateral hemispheres of sham-operated C4/− mice (Figure 6A; lanes Sham I and Sham C) C4 FL and bands corresponding to the C1 fragment do not differ in intensity but they are remarkably weaker compared with the ischemic areas I and C. There are differences of PrP full length and C1 signal intensities between ischemic *Prnp*^+/+^ and C4/− mice because of the 1.5 times higher PrP expression in C4/− mice (Figure 6B).

## DISCUSSION

In this study we first compared the lesion profile and extent of tissue damage in *Prnp*^+/+^ and *Prnp*^−/−^ mouse brains after transient focal ischemia. As shown before, *Prnp*^−/−^ mice have a tendency to larger infarct size when compared with *Prnp*^+/+^ animals (21, 31, 37). Our QRT-PCR analysis of *Prnp* gene expression after 1-h transient focal cerebral ischemia and 24-h reperfusion indicated distinct local differences in *Prnp*^+/+^ mouse brains. We found a decrease in *Prnp* expression in the lesion area while the infarct-free region around the lesion of the ipsilateral hemisphere showed a significant increase. A slight but nonsignificant increase of PrP^C^ mRNA levels was found in the contralateral hemisphere. The decrease of PrP^C^ mRNA levels in the necrotic area is readily explained by the large number of dying cells in this area. The up-regulation of PrP^C^ mRNA levels in the area adjacent to the infarct may be triggered by local oxidative damage and other metabolic derangements in this area; this would be in keeping with *in vitro* analyses showing a correlation between PrP expression and oxidative stress (3). The neuronal histology in the region of ischemic injury indicated oxidative damage and cell death. Intense PrP^C^ immunoreactivity was seen in individual cells in the penumbra, while other neurons in this area showed no PrP^C^ immunoreactivity.

The role of PrP^C^ FL in neuroprotection and in particular in reversing the oxidative stress damage is well documented (15, 23, 27, 39). In this study we detected an increased degradation of PrP^C^ FL to its breakdown product C1 in the area surrounding the infarct and in the contralateral hemisphere in *Prnp*^+/+^ mouse brains. This finding is in agreement with Mangé’s postulation of increased PrP^C^ degradation under oxidative conditions (20). It is tempting to speculate that PrP^C^ overexpression increases the chances for neuronal survival under the conditions of oxidative stress while lack of PrP^C^ facilitates cell death. However, the cascade may be more complex; volumetric analyses of mouse brains overexpressing PrP^C^ (tga20 line) did not show enhanced neuroprotection compared with wild-type control animals after ischemia (31). Disproportionate availability of downstream ligands may have an impact on the neuroprotective ability of PrP. A number of proteins, for example, the neuronal cell adhesion molecule (29), the stress-inducible protein 1 (19) or the laminin receptor and laminin receptor precursor protein (8, 11), have been postulated as potential interaction partners. It is also possible that PrP acts as a regulator of cytosolic signaling pathways. Increased activity of cell signaling factors involved in cell survival and apoptosis such as ERK-1/-2, STAT-1 and caspase-3 were found in *Prnp*^−/−^ mice (31, 38). At present it is difficult to decide whether the extent and severity of ischemia are a cause or a consequence of altered signaling pathways.

In previous cell culture studies we demonstrated that oxidative stress together with PrP^C^ elicited a signaling cascade involving PI3K (33). PI3K signaling increased the survival chances of PrP^C^-expressing cells. PI3K has been shown to be sensitive to redox signaling by superoxide and hydrogen peroxide and to be activated by copper ions (24). This effect may be mediated or initiated by PrP. In our experiments this interaction could not be initiated by PrP lacking the octapeptide and flanking amino acids (PrPΔ32–93). Other studies (36) have demonstrated that the octapeptide repeat region of PrP^C^ is required for reactive oxygen species-mediated β cleavage of PrP^C^ and cells lacking the octapeptide region are more susceptible to oxidative stress.

As our previous cell culture experiments pointed towards the neuroprotective effect of the N-terminal octapeptide repeat region (5) we were interested to investigate this effect *in vivo* using the established focal ischemia model. Therefore, we subjected transgenic C4/− mice to controlled ischemia by MCAO and reperfusion. C4/− mice lack the N-terminal octapeptide region (amino acids 51 to 91) and N-terminally flanking amino acids (PrPΔ32–93) and have been described as phenotypically normal under standard conditions but develop atypical disease when infected with scrapie prions (6). We show that in the focal ischemia model animals lacking the octapeptide of PrP (PrPΔ32–93) behave like *Prnp*^−/−^ mice. Thus, in our model the C4 protein (PrPΔ32–93) does not functionally rescue the *Prnp*^−/−^ phenotype. However, the precise mechanism of the PrP^C^-dependent neuroprotection remains to be elucidated.

On one hand studies have shown (28) that *Prnp*^−/−^ neuronal cells expressing PrP^C^ lacking the N-terminal octapeptide repeat region (amino acids 53–94) show enhanced apoptosis and decreased superoxide dismutase activity. In accordance with these findings Bounhar et al (2) demonstrated that PrP^C^ potently inhibits Bax-induced cell death in human primary neurons and while the deletion of four octapeptide repeats of PrP^C^ completely eliminates the neuroprotective effect of PrP^C^, PrP lacking the glycosylphosphatidylinositol anchor signal peptide remains anti-apoptotic.

On the other hand the N-terminal octapeptide region of PrP^C^ has been shown to bind copper in a cooperative manner (1, 26, 34). The most obvious train of arguments would invoke the copper-binding ability of PrP^C^ and its involvement in cell signaling, oxidative stress and the contribution to cell survival under stress conditions (33). PrP^C^ does not seem to be involved in copper transport at the synapse (10). Thus it would appear that PrP^C^ is a sensor for oxidative stress, which may function in conjunction with copper binding to the N-terminal octarepeat.

Sequence analysis by Chen et al (4) first detected a glycosylated, cell membrane-anchored major product, designated C1 that is generated by a cleavage at position 111/112 (α cleavage), as well as an additional fragment longer than C1, termed C2 which is created by β cleavage. These cleaving processes seem to be copper dependent (32). Interestingly, our Western blotting analyses showed that in C4/− mice there is an increase of the α cleavage in ischemic brain regions compared with sham-operated C4/− control animals. This finding indicates that the C1 fragment is not likely to be linked to the neuroprotective function of PrP^C^. Furthermore, in agreement with Watt and Hooper (36) no fragments at about 21.1 kDa corresponding to the C2 cleavage products were detectable in C4/− mice. As the β site is located in the octapeptide repeat region (22), the absence of a β cleavage in the octapeptide repeat region and its breakdown products in C4/− mice must be expected. In accordance with our data, McMahon et al (22) found a decrease of PrP^C^ FL and an increase of amino-terminally fragmented PrP^C^ in cells under oxidative stress. Furthermore they reported that the octapeptide repeat region of PrP^C^ undergoes a site-specific copper- and pH-dependent cleavage, which has vast implications for further proteolysis.

Mangé and coworkers (20) argued that PrP^C^ is first cleaved at the β site in the octapeptide region and near the histidine in position 96, followed by a rapid second cleavage in the α site, which inactivates the biological active form of PrP cleaved at the β site. This is in line with data of our experiments, where bands corresponding to the C2 fragments appeared to decrease in *Prnp*^+/+^ ischemic mouse brains, whereas an increase of bands corresponding to the C1 fragments was visible compared with *Prnp*^+/+^ sham controls. In mice expressing PrPΔ32–93 remarkably stronger bands representing the C1 fragments were detectable in ischemic brain areas compared with C4/− sham controls as well. Although C4/− mice express 1.5 times more PrP than wild-type mice and show an increase of C1 fragments under oxidative stress conditions, they fail to rescue the *Prnp*^−/−^ phenotype. Therefore our findings show that mice expressing PrPΔ32–93 were able to provide C1 terminal fragments independent of previous cleavage at the β site in the octapeptide region but the C1 fragments have no protective role.

Taken together our data support the hypothesis that absence of cleavage at the β site, which is possibly facilitated by copper binding, is associated with loss of PrP^C^ function.
